# The relationship between imaging features, therapeutic response, and overall survival in pediatric diffuse intrinsic pontine glioma

**DOI:** 10.1007/s10143-024-02435-8

**Published:** 2024-05-10

**Authors:** Xiaojun Yu, Mingyao Lai, Juan Li, Lichao Wang, Kunlin Ye, Dong Zhang, Qingjun Hu, Shaoqun Li, Xinpeng Hu, Qiong Wang, Mengjie Ma, Zeyu Xiao, Jiangfen Zhou, Changzheng Shi, Liangping Luo, Linbo Cai

**Affiliations:** 1 Department of Medical Imaging Center, Jinan University First Affiliated Hospital, No. 613, Huangpu Road West, Tianhe District, Guangzhou, 510630 Guangdong Province China; 2 Department of Oncology, Guangdong sanjiu Brain Hospital, No. 578, Shatai South Road, Baiyun District, Guangzhou, 510510 Guangdong Province China; 3 Department of Medical Imaging Center, The Fifth Affiliated Hospital of Jinan University, Yingke Avenue, Heyuan City, 517000 China

**Keywords:** Diffuse intrinsic pontine glioma (DIPG), Magnetic resonance imaging, Child, Prognosis, Radiotherapy

## Abstract

We aimed to evaluate the relationship between imaging features, therapeutic responses (comparative cross-product and volumetric measurements), and overall survival (OS) in pediatric diffuse intrinsic pontine glioma (DIPG). A total of 134 patients (≤ 18 years) diagnosed with DIPG were included. Univariate and multivariate analyses were performed to evaluate correlations of clinical and imaging features and therapeutic responses with OS. The correlation between cross-product (CP) and volume thresholds in partial response (PR) was evaluated by linear regression. The log-rank test was used to compare OS patients with discordant therapeutic response classifications and those with concordant classifications. In univariate analysis, characteristics related to worse OS included lower Karnofsky, larger extrapontine extension, ring-enhancement, necrosis, non-PR, and increased ring enhancement post-radiotherapy. In the multivariate analysis, Karnofsky, necrosis, extrapontine extension, and therapeutic response can predict OS. A 25% CP reduction (PR) correlated with a 32% volume reduction (R^2^ = 0.888). Eight patients had discordant therapeutic response classifications according to CP (25%) and volume (32%). This eight patients’ median survival time was 13.0 months, significantly higher than that in the non-PR group (8.9 months), in which responses were consistently classified as non-PR based on CP (25%) and volume (32%). We identified correlations between imaging features, therapeutic responses, and OS; this information is crucial for future clinical trials. Tumor volume may represent the DIPG growth pattern more accurately than CP measurement and can be used to evaluate therapeutic response.

**Supplementary Information:**

The online version contains supplementary material available at 10.1007/s10143-024-02435-8.

## Introduction

Diffuse intrinsic pontine gliomas (DIPGs) account for 80% of all brainstem gliomas and 10–20% of all central nervous system tumors in children [9]. DIPGs are pediatric malignant brainstem tumors that lead to a median survival time of less than 1 year [11]. The anatomical complexity and critical functions of the brainstem preclude surgical resection. Radiation therapy, the standard of care [7], which can prolong 3-to 4-month survival time. The management strategies other than local irradiation are ineffective [2]. Recent studies have shown that reirradiation can prolong the survival time in children with DIPG [1, 13, 15].

In recent years, DIPG biology has been studied comprehensively, and these tumors have been found to be largely characterized by a mutation in the genes encoding histones H3F3A and HIST1H3B; in the new 2021 WHO classification, diffuse midline gliomas were renamed to diffuse midline gliomas H3K27-altered [18]. Imaging is essential for reaching a diagnosis of DIPG, defining the extent of the tumor, and assessing therapeutic response or disease progression (including subsequent imaging examinations conducted in trials). In recent years, some studies have explored the relationship between the clinical and imaging characteristics of DIPG and overall survival (OS) [12, 17]. Most previous studies were based on two-dimensional (2D) measurements and imaging characteristics of tumors. Recently, the Response Assessment in Pediatric Neuro-Oncology (RAPNO) working group provided tailored recommendations for assessing therapeutic responses in DIPG [3]. However, DIPGs are invasive tumors, which usually have irregular boundaries, and most of these tumors have an extrapontine extension [11, 17]; therefore, 2D measurements may not be able to accurately evaluate some imaging features and treatment responses.

A recent study reported that volumetric assessments in patients with progressive disease (PD) correlated more strongly with survival than the 2D measurements at most timepoints [16]. However, there are few reports on the relationship between therapeutic response and OS; one study showed that patients with a tumor volume decrease of more than 25% during radiotherapy had longer OS [21]. Therefore, this study aimed to explore the relationship between imaging features, therapeutic responses, and OS in pediatric DIPG. Furthermore, we aimed to assess the correlation between cross-product (CP) and DIPG volume assessment results and relationship between therapeutic response and OS in DIPG patients by comparing CP and volumetric measurements; we considered that our findings would be likely to be critical for future clinical trials and for the improved detection of therapeutic effects.

## Materials and methods

### Study population

The institutional review committee of our center approved this retrospective study and waived the requirement for informed consent.

**Fig. 1:**
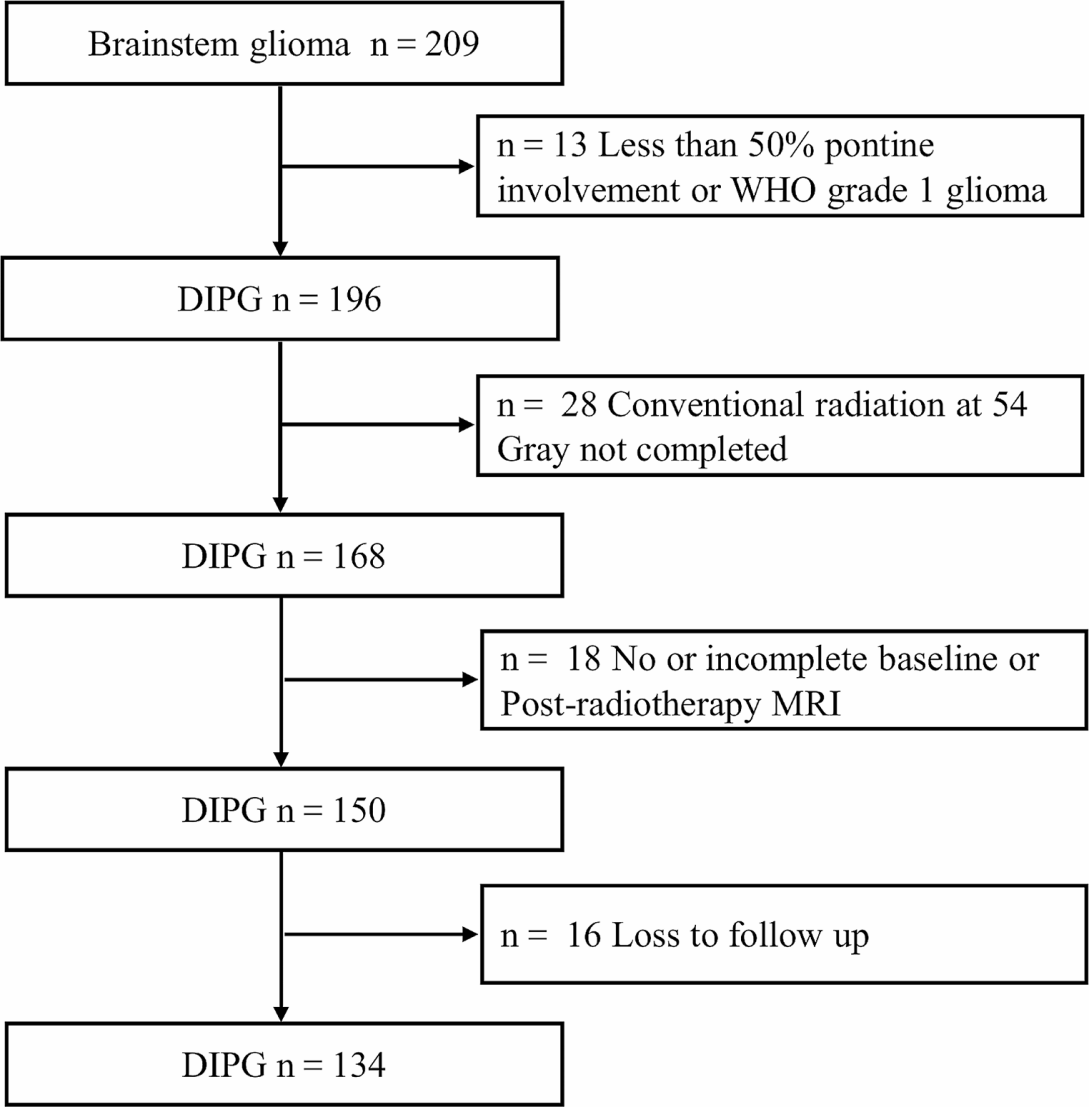
Flowchart of patients excluded from this study DIPG, diffuse intrinsic pontine glioma

From December 2010 to February 2023, 209 consecutive patients aged ≤ 18 years who were diagnosed with brainstem glioma through MRI were identified. Among these, 75 patients were excluded due to multiple reasons (Fig. 1).

All the patients received a conventional radiation dose of 54 Gy in 30 fractions (f). All patients underwent brain MRI examinations within 3 weeks before radiotherapy and 4–6 weeks post-radiotherapy. OS was defined as the time from the date of diagnosis to the date of death.

### Clinical variables

Clinical information was obtained from the patients’ medical records and included details regarding age, sex, time of DIPG diagnosis, Karnofsky at diagnosis, radiotherapy, symptoms, and changes in symptoms.

### MRI parameters

MR images were obtained using a 3.0-T or 1.5-T MR scanner with an 8-channel head coil. Imaging sequences included axial T1WI, T2WI, T2 fluid-attenuated inversion recovery (FLAIR), sagittal T1WI, and contrast-enhanced T1WI (axial, sagittal, and coronal). The following parameters were used for T1WI: TR, 488–1900 ms, TE, 15–28 ms; T2WI: TR, 3980–4480 ms, TE, 110–120 ms; T2-FLAIR: TR, 6000–9480 ms, TE, 120–135 ms; field of view, 240 × 240 mm; 256 × 256 matrix; slice thickness, 5/5.5 mm (with a gap of 0/1 mm).

### Imaging evaluation

**Fig. 2:**
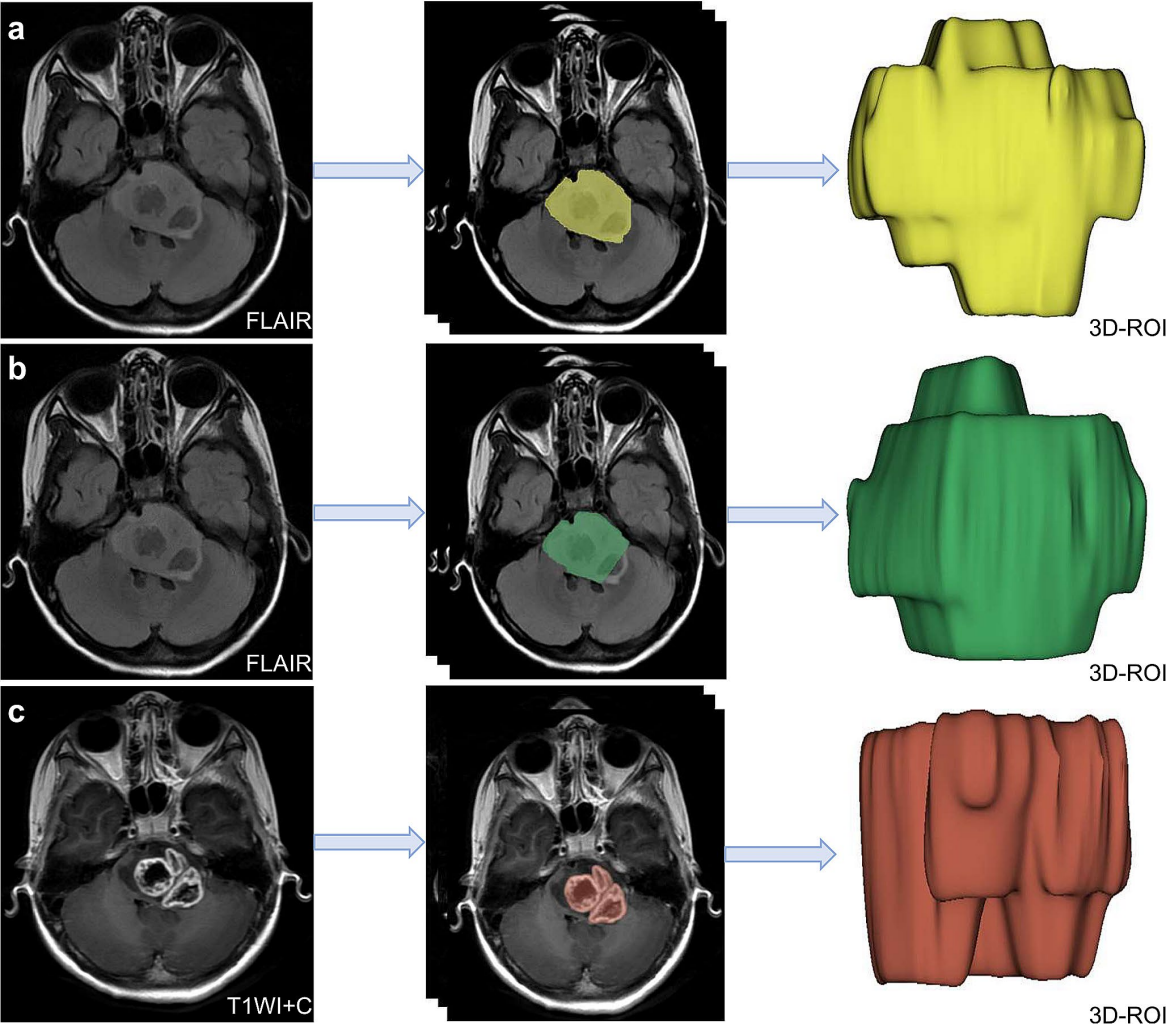
Volume measurements. (**a-b**). Axial T2-FLAIR images used to measure total tumor volume (yellow) and pontine tumor volume (green) in DIPG. (**c**). Axial T1WI + C used to measure ring enhancement volume (red) in DIPG. Extrapontine tumor volume was defined as total tumor volume minus pontine tumor volume DIPG, diffuse intrinsic pontine glioma; FLAIR, fluid-attenuated inversion recovery; T1WI + C, contrast-enhanced T1WI; 3D-ROI, three-dimensional region of interest

Sagittal T1WI images together with axial T2-FLAIR images were used to measure the craniocaudal tumor dimensions. Axial T2-FLAIR images together with sagittal T1WI images were used to obtain the 2D product of the largest perpendicular diameters and volume measurements. The 2D measurements were performed using the offline software application, the RadiAnt DICOM Viewer (Medixant; RadiAnt DICOM Viewer Software, Version 2021.1; Jun 27, 2021; URL: https://www.radiantviewer.com). Total tumor volume, pontine tumor volume, and ring enhancement were measured using 3D Slicer (http://www.slicer.org), as reported in the study by Makepeace et al. [19]. (Fig. 2).

All the MR images were reviewed by two observers (with 8 and 10 years of radiological imaging experience, respectively). For quantitative parameters, such as tumor craniocaudal size, tumor CP size, tumor volume, pontine lesion volume, and ring enhancement volume, the average of the values measured by the two observers was used as the final value. Imaging features, including ring enhancement and necrosis, were evaluated visually. Necrosis was defined as the area with a clear boundary and liquid-like signal in the tumor rather than the non-enhancement area with an indistinct boundary (for example, T2-FLAIR mismatch signal); contrast enhancement was mostly ring enhancement (Supplementary).

### Definitions of therapeutic response

The effect of radiotherapy on the tumors was determined according to the DIPG RAPNO criteria [3]. Partial response (PR) was defined as a ≥ 25% decrease (compared with baseline) in the tumor 2D product of perpendicular diameters. PD was defined as a ≥ 25% increase (compared with baseline) in the 2D product of perpendicular diameters. The response was classified as stable disease (SD) if it did not meet the criteria for PR or PD. Patients who were initially assessed as PD were re-evaluated within 4–8 weeks exclude pseudoprogression.

### Statistical analysis

Statistical analyses were performed using SPSS (version 27.0; IBM Corp) and R software (version 4.2.0, www.R-project.org). Univariate and multivariate analyses of imaging characteristics, clinical data, therapeutic responses, and OS were performed using Cox proportional hazards regression. A nomogram was established based on the results of the multivariate analysis. OS was estimated using the Kaplan-Meier method. A Pearson correlation analysis was used to compare the correlations between baseline and post-radiotherapy CP and volume. To determine the volume threshold corresponding to the CP threshold (25% decrease) in case of PR, linear regression was used to compare the percentage change in tumor size between the CP and volumetric methods at baseline and post-radiotherapy. The log-rank test was used to compare OS between patients with a discordant therapeutic response classification according to the CP and volume measurements and those with a concordant classification according to CP and volume. The intraclass correlation coefficient (ICC) was used to evaluate the interobserver agreement in the following measurements: CP size, craniocaudal size, tumor volume, pontine lesion volume, and ring enhancement volume. Statistical significance was set at *P* < 0.05.

## Results

### Demographic and clinical characteristics and OS

This study included 134 patients with DIPGs. The follow-up period ended in January 2024; 129 patients died; 5 patients survived. The median survival time was 10.4 months. The 12- and 24- month OS rates were 38.06% and 2.24%, respectively. The demographic information is summarized in Table 1.

**Table 1:** Baseline and post-radiotherapy imaging, clinical, and therapeutic features

Characteristic	DIPG (*n* = 134)
**Clinical**	
Age (years)	2–16
Sex	
Male	69 (51.49%)
Female	65 (48.51%)
Survival condition	
Death	129 (96.27%)
Overall survival (months)	4.00–29.30
Median survival (months)	10.40
12- month overall survival rates	38.06%
24- month overall survival rates	2.24%
**Imaging Feature**	
**Baseline**	
Cross product (cm^2^)	20.83 ± 5.21
Volume (ml)	46.25 ± 15.76
Craniocaudal dimension (cm)	4.46 ± 0.86
Necrosis	55 (41.04%)
Extrapontine lesion extension	125 (93.28%)
Extrapontine lesion extension ratio (×100%)	18.52 ± 9.98
Ring enhancement	52 (38.81%)
Karnofsky score	65.0 (50.00–70.00)
**Post-radiotherapy**	
Cross product (cm^2^)	15.65 (11.09–20.45)
Volume (ml)	31.02 (19.37–45.27)
Increased ring enhancement	54 (40.30%)
Worsened symptoms	16 (11.94%)
Increased ring enhancement and worsened symptoms	13 (9.70%)

Abbreviations: DIPG, diffuse intrinsic pontine glioma; Gy, gray

**Fig. 3:**
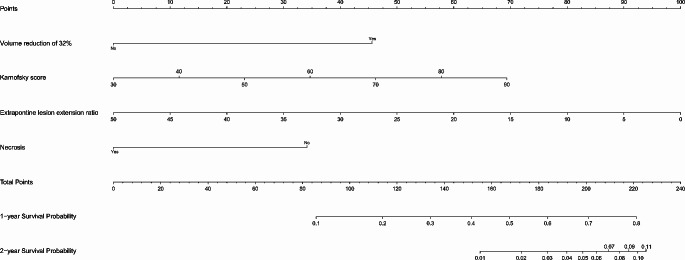
The 1-year and 2-year nomogram for survival probability of patients with DIPG DIPG, diffuse intrinsic pontine glioma

The interobserver ICCs corresponding to tumor craniocaudal size, tumor CP, tumor volume, pontine lesion volume, and ring enhancement volume were 0.936, 0.897, 0.983, 0.946, and 0.986, respectively. The 95% confidence intervals (CI) are summarized in Table 2. The baseline and post-radiotherapy imaging and clinical features are summarized in Table 1. The results of the univariate analysis of clinical and imaging features, therapeutic responses, and OS are summarized in Table 3. The results of the multivariable analyses of clinical and imaging features, therapeutic response-related imaging findings, and OS are summarized in Table 3. According to the multivariate Cox regression results, the 1-year and 2-year OS rate nomograph is established, as shown in Fig. 3.

**Table 2:** Interobserver agreement of DIPG quantitative parameters

Measurement	ICC (95% CI)
Tumor Craniocaudal size (cm)	0.936 (0.909, 0.955)
Tumor Cross product (cm^2^)	0.897 (0.868, 0.920)
Tumor volume (cm^3^)	0.983 (0.978, 0.987)
Pontine lesion volume (cm^3^)	0.946 (0.922, 0.963)
Ring enhancement volume (cm^3^)	0.986 (0.979, 0.991)

Abbreviations: DIPG, diffuse intrinsic pontine glioma; ICC, intraclass correlation coefficient; CI, confidence interval

**Table 3:** Univariate and multivariate analyses of the correlations of clinical and imaging features and therapeutic responses with overall survival

Variable	HR	*P* value
**Univariate**		
Age (continuous)		0.064
Age (Categorical)		0.106
≤ 3		
> 3		
Karnofsky score	0.97 (0.96–0.98)	< 0.001^***^
Tumor CP Size		0.296
Tumor volume		0.256
Craniocaudal dimension		0.366
Necrosis (55/134)	1.72 (1.20–2.46)	0.003^**^
Ring enhancement (52/134)	1.69 (1.18–2.43)	0.004^**^
Extrapontine lesion extension ratio (× 100%)	1.04 (1.02–1.06)	< 0.001^***^
Therapeutic response (CP reduction of 25%)		< 0.001^***^
PR (n = 63)	1.00	
Non-PR (SD (n = 64)/PD (n = 7))	2.27 (1.57–3.27)	
Therapeutic response (volume reduction of 32%)		< 0.001^***^
PR (n = 71)	1.00	
Non-PR (SD (n = 56)/PD (n = 7))	2.50 (1.74–3.61)	
Post-radiotherapy increased ring enhancement (54/134)	2.16 (1.50–3.10)	< 0.001^***^
**Multivariate**		
Karnofsky score	0.97 (0.96–0.99)	< 0.001^***^
Necrosis	1.55 (1.08–2.23)	0.017^*^
Extrapontine lesion extension ratio (× 100%)	1.03 (1.01–1.05)	0.006^**^
Therapeutic response (volume reduction of 32%)		0.008^**^
PR	1.00	
Non-PR (SD/PD)	1.73 (1.15–2.61)	

^*^P < 0.05, ^**^P < 0.01, ^***^P < 0.001

Abbreviations: PR, partial response; SD, stable disease; PD, progressive disease; CP, cross-product; HR, hazards ratio

**Fig. 4:**
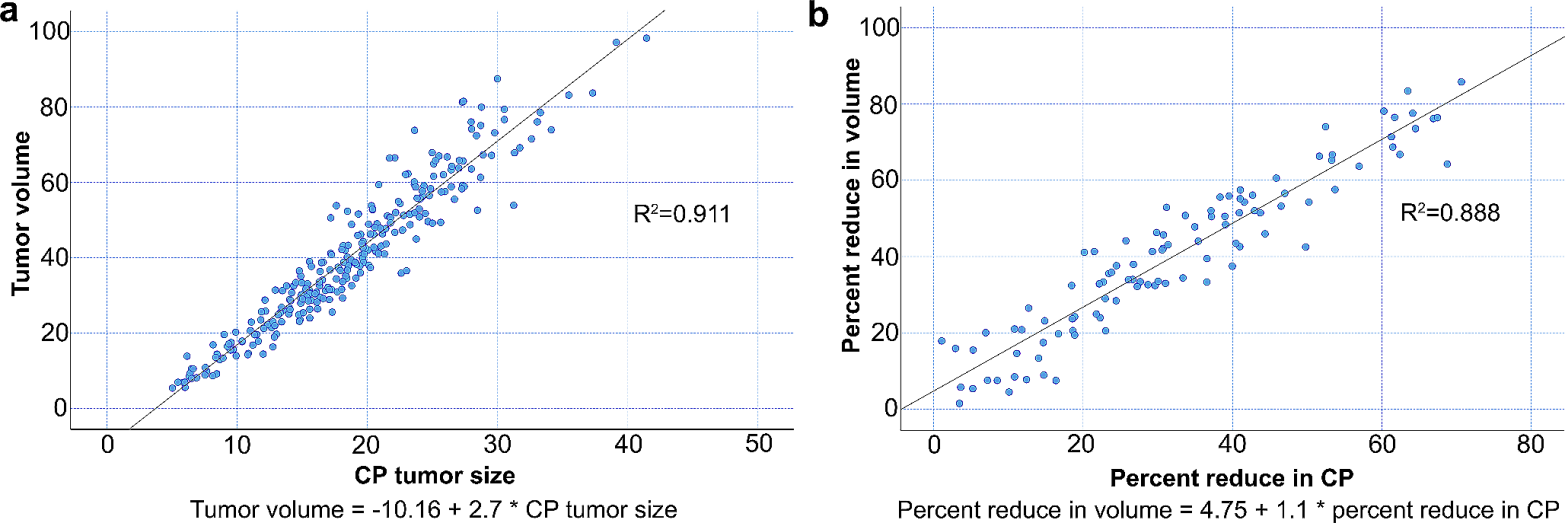
(**a**) Correlation between DIPG cross product and volume at 268 timepoints (baseline and post-radiotherapy) in 134 patients. (**b**) Correlation between DIPG cross-product and volume reduction in 102 patients (baseline and post-radiotherapy) DIPG, diffuse intrinsic pontine glioma; CP, cross-product

**Fig. 5:**
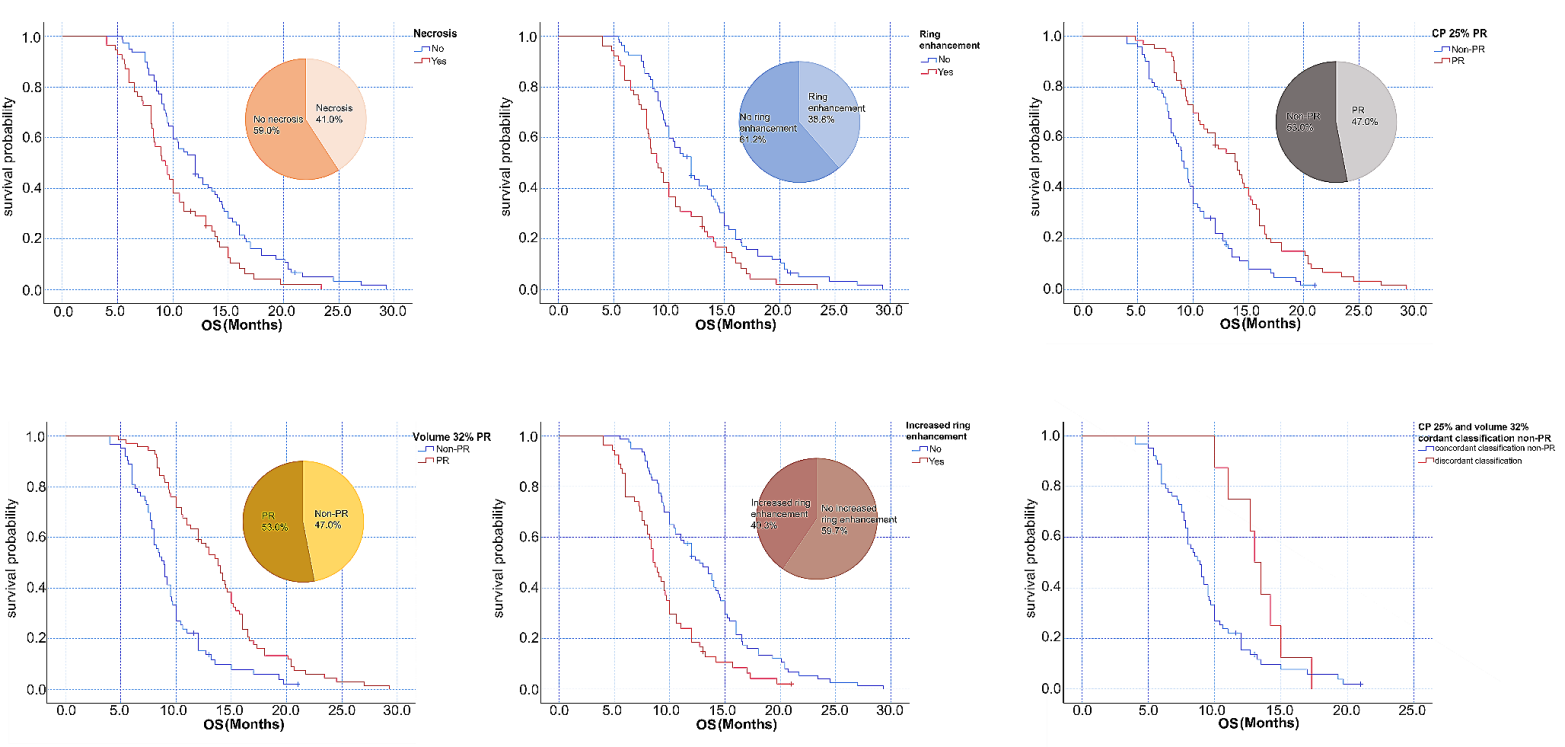
Kaplan-Meier curves for overall survival (OS) based on (**a**) necrosis, (**b**) ring enhancement, (**c**) CP-based therapeutic response (definition of PR: CP reduction of 25%), (**d**) volume-based therapeutic response (definition of PR: volume reduction of 32%), and (**e**) post-radiotherapy increased ring enhancement. **f.** Kaplan-Meier survival curves showing a comparison between patients with a discordant classification (CP reduction of 25% and volume reduction of 32%) and those with a concordant classification in the non-PR group CP, cross-product; PR, partial response; increased ring enhancement, post-radiotherapy increased ring enhancement

A total of 268 CP and volume measurements were performed in 134 patients at baseline and post-radiotherapy. The CP and volume measurements were strongly correlated (R^2^ = 0.911), as shown in Fig. 4a. Among 134 patients, 102 patients exhibited a simultaneous reduction in CP and volume post-radiotherapy. Linear regression analysis showed that PR based on CP (25% decrease) corresponded to a decrease of approximately 32% in segmented volume (R^2^ = 0.888), as shown in Fig. 4b. The results of the Kaplan-Meier survival analyses of necrosis, ring enhancement, therapeutic response, and post-radiotherapy increased ring enhancement are shown in Fig. 5a-e.

**Fig. 6:**
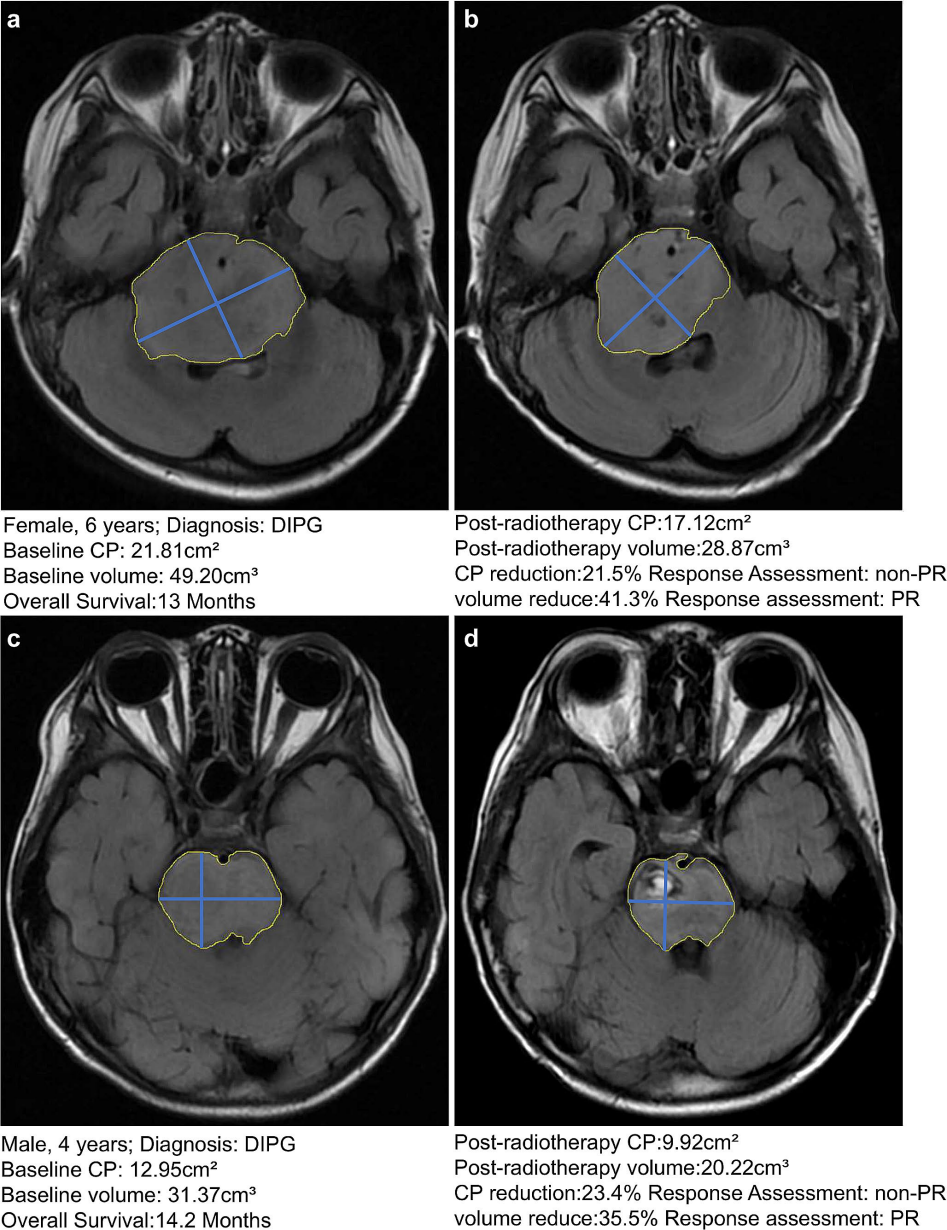
Representative case of PR with a discordant classification based on a CP reduction of 25% and volume reduction of 32%. (**a-b**). A 6-year-old female DIPG patient with an overall survival (OS) of 13 months. Measurement of the 2D product of the maximum peripheral diameter and tumor volume at baseline and post-radiotherapy in T2-FLAIR images. The CP and volume measurements showed that the tumor exhibited a 21.5% CP reduction and 41.3%, volume reduction post-radiotherapy. Non-PR and PR classifications based on CP and volume response assessments, respectively. (**c-d**). A 4-year-old female DIPG patient with an overall survival (OS) of 14.2 months. Measurement of the 2D product of the maximum peripheral diameter and tumor volume at baseline and post-radiotherapy in T2-FLAIR images. The CP and volume measurements showed that the tumor exhibited a 23.4% CP reduction and 35.5% volume reduction post-radiotherapy. Non-PR and PR classifications based on CP and volume response assessments, respectively DIPG, diffuse intrinsic pontine glioma; FLAIR, fluid-attenuated inversion recovery; CP, cross-product; PR, partial response; 2D, two-dimensional

The median survival time based on CP (25%) and volume (32%) in the PR group was 14.0 months and 13.8 months, respectively, while that in the non-PR group was 9.2 and 8.9 months, respectively. The response in a total of eight patients was classified as SD according to CP (25%) and as PR according to volume (32%), median survival time was 13.0 months, which was significantly higher than that in the non-PR group (8.9 months), in which the therapeutic response was consistently classified as non-PR based on CP (25%) and volume (32%) (*P* = 0.039) (Fig. 5f). Representative case of PR with a discordant classification based on a CP reduction of 25% and volume reduction of 32% is shown in Fig. 6.

Among the 134 patients, 16 (11.94%) exhibited worse symptoms post-radiotherapy, of whom 13 patients showed increased ring enhancement post-radiotherapy.

## Discussion

In this 10-year single-center study, we evaluated the survival outcomes, as well as the clinical, radiological, and therapeutic response-related factors that affect prognosis, in children with DIPG who received radiation therapy. Our findings confirmed some previously reported survival-related factors in DIPG patients [11, 12, 14, 17, 21], such as extrapontine lesion extension, necrosis, ring enhancement, Karnofsky, therapeutic response, and increased ring enhancement post-radiotherapy. Our results showed that 39% of patients exhibited ring enhancement, which is generally the same as previously reported results, and the proportion of patients with ring enhancement was approximately 35–37% [11, 12, 17].

Most previous studies [11, 14, 17] used visual examinations or 2D measurements to evaluate the extension of extrapontine lesions. In this study, we found that a three-dimensional (3D) quantitative measurement of the extrapontine extension can be used to evaluate the various forms and patterns of extrapontine extensions of tumors more accurately. We found that a higher Karnofsky at diagnosis was associated with longer OS, which is consistent with the results of a previous study [14, 26]. This may be because patients with high Karnofsky can receive more active treatment, which leads to some benefits. Previous studies have shown that larger craniocaudal tumor dimensions are significantly associated with shorter OS [11, 17]. Our results showed that tumor size and craniocaudal tumor dimensions had no significant relationship with OS, which is consistent with the findings of previous studies on DIPG [8, 23].

A response assessment of brain tumors is highly dependent on radiological criteria [5], and accurate reproducible assessments are crucial for determining the therapeutic response; the RAPNO working group has recommended the use of the 2D product of the largest perpendicular diameters in DIPG studies [3]. However, DIPG tumors have irregular shapes and most of them have extrapontine lesion extensions, which presents some challenges [24]. It is difficult to obtain reliable and consistent tumor measurements, even when performed by an experienced imaging reader [10].

In recent years, a growing number of studies have shown that volumetric measurements may be more accurate than 2D methods for assessing tumor growth [4, 6, 20–22]. A recent study results showed that volumetric assessments in PD correlated more strongly with survival than the 2D measurements at most timepoints [16]. A previous study has shown that patients with a tumor volume decrease of > 25% during radiotherapy had longer OS [21]. However, these previous studies did not directly compare the threshold of volume reduction corresponding to a 25% reduction in CP. Therefore, this study further explored the threshold of volume reduction corresponding to a 25% reduction in CP to perform a comparison. The volume segmentation method can be used to measure DIPG volume changes before and after therapy and the volume of tumors originating from the pons more accurately. In addition, this consistent and limited location of the tumors may strengthen the relationship between tumor volume change and therapeutic response. Consequently, it was possible to explain the non-spherical DIPG growth pattern further [6].

In eight (6.0%) of the 134 patients, we observed discordant results between CP (25%) and volume (32%). Their median survival time was 13.0 months, which was significantly higher than that of the non-PR group (8.9 months). Therefore, it is more reasonable to classify the response in these eight patients as PR according to the volume (32%) criteria.

Given the limitations of 2D tumor measurements, the use of a volumetric assessment would allow for a more accurate determination of the contrast-enhancing volumes [25]. Another important finding in this study is that the volume change on comparing ring enhancement before and post-radiotherapy was closely related to survival, which is manifested in the shorter OS in patients with increased ring enhancement volume post-radiotherapy. Previous studies have mostly explained this as radiation-induced necrosis [7]. Therefore, when the clinical symptoms of patients worsen, MRI should be performed in a timely manner, and the ring enhancement of lesions should be assessed, which would be helpful in choosing a more appropriate time for using antiangiogenic drugs.

Our study had some limitations. First, due to the low incidence of DIPG, the sample size in this retrospective study was relatively small. The next step is to expand our dataset. Second, our study from a single institution, therefore, a multicenter prospective study is required to verify our results. Finally, there were limited molecular data for analysis in this study, we were unable to further analyze the molecular data.

## Conclusions

This study shows that determining the correlations between imaging features, therapeutic responses, and OS is crucial for further risk stratification of patients and for guidance in clinical decision-making in future clinical trials. In addition, tumor volume measurement may represent the tumor growth pattern more accurately than CP measurement and can be used to evaluate the therapeutic response in patients with DIPG.

## Electronic supplementary material

Below is the link to the electronic supplementary material.

Supplementary Material 1

## Data Availability

No datasets were generated or analysed during the current study.

## Abbreviations

DIPG: Diffuse intrinsic pontine gliomas
OS: Overall survival
CP: Cross-product
PR: Partial response
2D: Two-dimensional
RAPNO: Response Assessment in Pediatric Neuro-Oncology
PD: Progressive disease
Gy: Gray
FLAIR: Fluid-attenuated inversion recovery
SD: Stable disease
ICC: Intraclass correlation coefficient
CI: Confidence interval
3D: Three-dimensional
