# Correction to: The relationship between imaging features, therapeutic response, and overall survival in pediatric diffuse intrinsic pontine glioma

**DOI:** 10.1007/s10143-024-02484-z

**Published:** 2024-06-10

**Authors:** Xiaojun Yu, Mingyao Lai, Juan Li, Lichao Wang, Kunlin Ye, Dong Zhang, Qingjun Hu, Shaoqun Li, Xinpeng Hu, Qiong Wang, Mengjie Ma, Zeyu Xiao, Jiangfen Zhou, Changzheng Shi, Liangping Luo, Linbo Cai

**Affiliations:** 1 Department of Medical Imaging Center, Jinan University First Affiliated Hospital, Tianhe District, No. 613, Huangpu Road West, Guangzhou, 510630 Guangdong Province China; 2 Department of Oncology, Guangdong sanjiu Brain Hospital, No. 578, Shatai South Road, Baiyun District, Guangzhou, 510510 Guangdong Province China; 3 Department of Medical Imaging Center, The Fifth Affiliated Hospital of Jinan University, Yingke Avenue, Heyuan City, 517000 China

**Correction to: Neurosurgical Review**

10.1007/s10143-024-02435-8

The authors regret that the version of Table 3 that appears in the original published article contains some errors.

The incorrect Table 3 appears below.

Table 3. Univariate and multivariate analyses of the correlations of clinical and imaging features and therapeutic responses with overall survival

**Table:** 

Variable	HR	*P* value
Univariate		
Age (continuous)		0.064
Age (Categorical)		0.106
≤ 3		
> 3		
Karnofsky score	0.97 (0.96–0.98)	< 0.001^***^
Tumor CP Size		0.296
Tumor volume		0.256
Craniocaudal dimension		0.366
Necrosis (55/116)	1.72 (1.20–2.46)	0.003^**^
Ring enhancement (52/116)	1.69 (1.18–2.43)	0.004^**^
Extrapontine lesion extension ratio (× 100%)	1.04 (1.02–1.06)	< 0.001^***^
Therapeutic response (CP reduction of 25%)		< 0.001^***^
PR (*n* = 63)	1.00	
Non-PR (SD (*n* = 64)/PD (*n* = 7))	2.27 (1.57–3.27)	
Therapeutic response (volume reduction of 32%)		< 0.001^***^
PR (*n* = 71)	1.00	
Non-PR (SD (*n* = 56)/PD (*n* = 7))	2.50 (1.74–3.61)	
Post-radiotherapy increased ring enhancement (54/134) (42/108+)	2.16 (1.50–3.10)	< 0.001^***^
Multivariate		
Karnofsky score	0.97 (0. 96–0.99)	< 0.001^***^
Necrosis	1.55 (1.08–2.23)	0.017^*^
Extrapontine lesion extension ratio (× 100%)	1.03 (1.01–1.05)	0.006^**^
Therapeutic response (volume reduction of 32%)		0.008^**^
PR	1.00	
Non-PR (SD/PD)	1.73 (1.15–2.61)	

^*^ *P* < 0.05, ^**^ *P* < 0.01, ^***^ *P* < 0.001.

Jiangfen ZhouAbbreviations: PR, partial response; SD, stable disease; PD, progressive disease; CP, cross-product; HR, hazards ratio 

The correct Table 3 is shown below.

Table 3. Univariate and multivariate analyses of the correlations of clinical and imaging features and therapeutic responses with overall survival

**Table:** 

Variable	HR	*P*** value**
Univariate		
Age (continuous)		0.064
Age (Categorical)		0.106
≤ 3		
> 3		
Karnofsky score	0.97 (0.96–0.98)	< 0.001^***^
Tumor CP Size		0.296
Tumor volume		0.256
Craniocaudal dimension		0.366
Necrosis (55/134)	1.72 (1.20–2.46)	0.003^**^
Ring enhancement (52/134)	1.69 (1.18–2.43)	0.004^**^
Extrapontine lesion extension ratio (× 100%)	1.04 (1.02–1.06)	< 0.001^***^
Therapeutic response (CP reduction of 25%)		< 0.001^***^
PR (*n* = 63)	1.00	
Non-PR (SD (*n* = 64)/PD (*n* = 7))	2.27 (1.57–3.27)	
Therapeutic response (volume reduction of 32%)		< 0.001^***^
PR (*n* = 71)	1.00	
Non-PR (SD (*n* = 56)/PD (*n* = 7))	2.50 (1.74–3.61)	
Post-radiotherapy increased ring enhancement (54/134)	2.16 (1.50–3.10)	< 0.001^***^
Multivariate		
Karnofsky score	0.97 (0. 96–0.99)	< 0.001^***^
Necrosis	1.55 (1.08–2.23)	0.017^*^
Extrapontine lesion extension ratio (× 100%)	1.03 (1.01–1.05)	0.006^**^
Therapeutic response (volume reduction of 32%)		0.008^**^
PR	1.00	
Non-PR (SD/PD)	1.73 (1.15–2.61)	

* *P <* 0.05, ** *P <* 0.01, *** *P <* 0.001.

PR, partial response; SD, stable disease; PD, progressive disease; CP, cross-product; HR, hazards ratio

The original article has been corrected.

