# Supplementary material for: The relationship between imaging features, therapeutic response, and overall survival in pediatric diffuse intrinsic pontine glioma
Source: Neurosurg Rev. 2024 May 10;47(1):212. doi: 10.1007/s10143-024-02435-8 (PMC11087318; doi:10.1007/s10143-024-02435-8)
Supplement: Supplementary file 1 — Supplementary Material 1 [file 10143_2024_2435_MOESM1_ESM.docx]

**Supplementary material:**

**Definition of necrosis**

Necrosis was defined as the area with a clear boundary and liquid-like signal in the tumor rather than the non-enhancement area with an indistinct boundary (for example, T2-FLAIR mismatch signal); contrast enhancement was mostly ring enhancement.
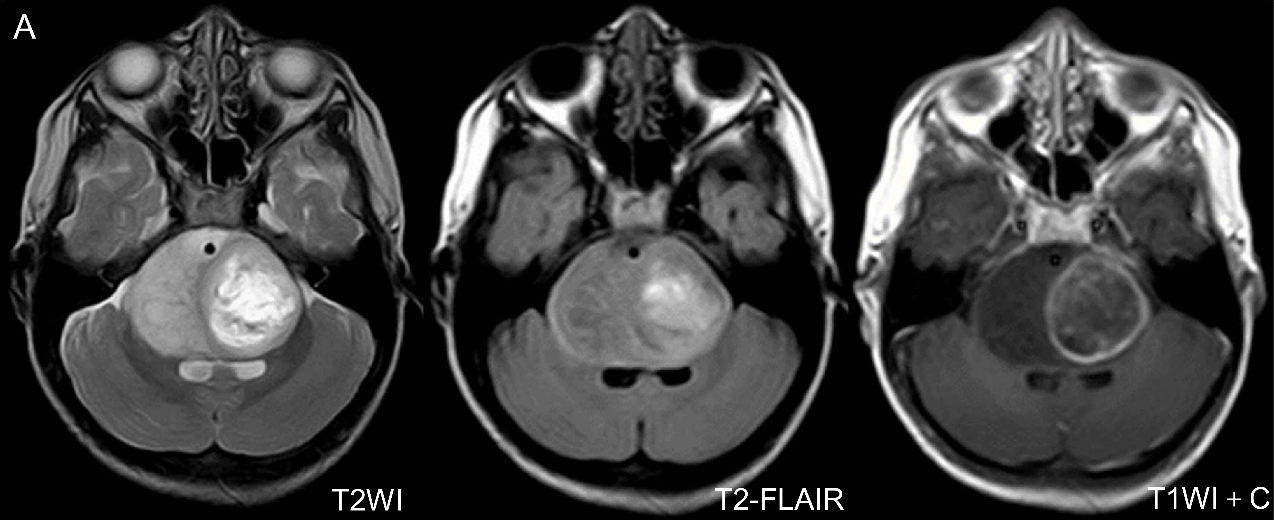


Figure A. Necrosis areas. T2WI showed the clear boundary of the necrosis area and fluid-like signal present in the tumor, and an annular low signal was seen surrounding the area. T2-FLAIR images showed patchy high signal intensity (hemorrhage); in most of the necrosis areas, peripheral rim-like enhancement was observed on administering the contrast agent. A visual evaluation showed that most of the necrosis areas in the cross section did not exceed two-third of the whole tumor area.





Figure B. T2-FLAIR mismatch signal. T2 weighted (“high intensity”) and FLAIR (“low intensity”) signal; no enhancement was seen when the contrast agent was administered. The T2-FLAIR mismatch signal area in the visual evaluation cross section usually exceeded two-third of the whole tumor area.
